# Animal Temperature

**Published:** 1848-05

**Authors:** 


*Animal Temperature.*—M. C. Dr. Demarquay, Demonstrator to the Faculty of Medecine of Paris, has made a series of researches and experiments on animal temperature which seems to lead to the following conclusions. When an animal suffers from severe pain its temperature is augmented; hæmorrhage, which is speedily fatal, but little modifies this function. He tied the aorta and inferior cava, the arteries and veins of the extremities, and the carotids, at different times, and the result of his experiments was that the ligature of veins lowered the temperature less than that of the arteries; that while a ligature on the abdominal aorta lowered the temperature of the body 8 or 10 degrees in a few hours, a ligature on the femoral artery only reduced the heat of the limb one or two degrees. M. Demarquay found that inflamed wounds increased the local and general temperature, but the former, when the inflamed part is at a distance from the centre of the circulation, never equals the general or natural temperature as taken by a thermometer in the rectum. He found that tying the vessels going to an inflamed part did not check the increase of heat in that part. A number of animals were sacrificed in experiments to determine the effects of internal strangulations in modifying the temperature of the body, and it was found that their occurrence quickly reduced the general temperature, and that this reduction was greater when a portion of intestine was tied, so as that its whole circumference was not included in the ligature, than when the thread was tied circularly on it, and also that the reduction of temperature was great in proportion as the strangulated bowel was near the stomach. M. Demarquay and M. Duméril, jr., likewise made experiments together on the effects of certain poisons on temperature, and found that some quickly raised it, as digitalis, belladonna, croton oil and strychnine; others, on the contrary, reduced it, as cyanide of potassium, corrosive sublimate, arsenious acid, tartar emetic, muriate of morphia, and ammonia, &c., and that etherization carried to a great extent, sensibly lowers the animal temperature, and that this reduction of heat commences immediately that insensibility occurs, and continues to increase to the animal's death.—*Dublin Medical Press.*

